# P-1433. Direct vs. Indirect Effects of Influenza Vaccination on Cardiovascular Risk: A Causal Mediation Study

**DOI:** 10.1093/ofid/ofaf695.1620

**Published:** 2026-01-11

**Authors:** Alexia El Khoury, Ethan Martin, Chris D Ladikos, Zainab Albar, Joy Abou Farah, Jay Krishnan, Elie Saade

**Affiliations:** Case Western Reserve university, Cleveland, OH; Case Western Reserve university, Cleveland, OH; University hospitals, Cleveland, Ohio; Case western reserve university school of medicine, cleveland, Ohio; Case Western Reserve university, Cleveland, OH; University hospitals, ID clinical trial unit, Cleveland, Ohio; Case Western Reserve University, Cleveland, OH

## Abstract

**Background:**

Influenza infection is a well-described trigger for major adverse cardiovascular events (MACE), particularly in older adults. While influenza vaccination has been associated with reduced cardiovascular risk, it is unknown whether the benefit is primarily due to infection prevention, attenuation of disease severity, or other biological or behavioral mechanisms. This study applies a novel mediation analysis to untangle the pathways by which influenza vaccination may reduce MACE.

**Figure:**
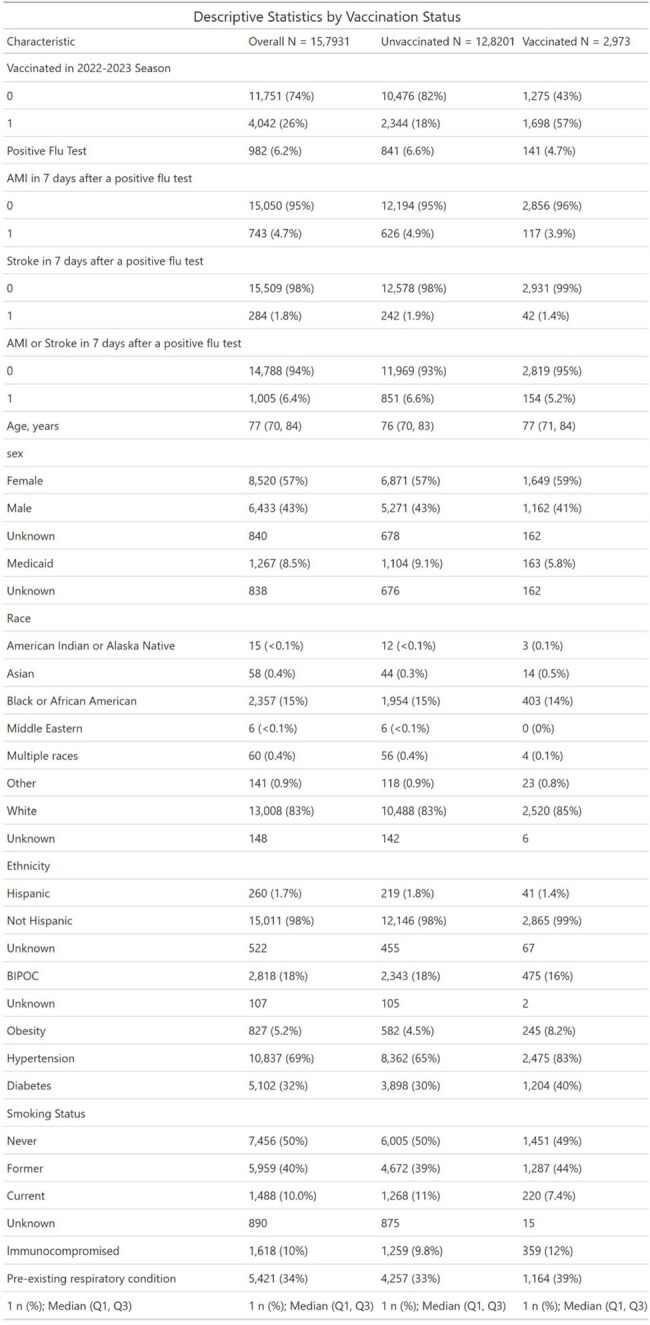
Descriptive statistics by vaccination status

**Figure:**
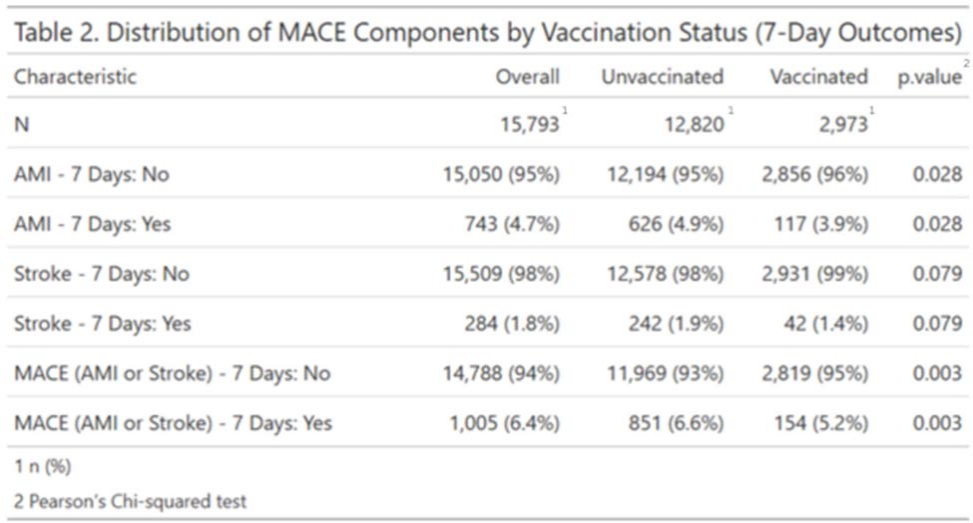
Distribution of AMI, Stroke, and Composite MACE Events Within 7 Days by Vaccination Status. This table summarizes the incidence of acute myocardial infarction (AMI), stroke, and composite major adverse cardiovascular events (MACE) within 7 days following influenza testing, stratified by vaccination status. Results highlight lower event rates among vaccinated individual

**Methods:**

We conducted a retrospective cohort study of adults aged ≥ 65 with at least one PCR flu test during the 2023–2024 influenza season. Vaccination status, the primary exposure variable, and laboratory-confirmed influenza infection were identified through EHR data. The primary outcome, MACE was defined as any diagnosis of acute myocardial infarction (AMI) or stroke by ICD-10 codes, within 7 days of influenza testing. Causal mediation analysis using a counterfactual framework were used to estimate the total effect of vaccination on MACE and its decomposition into indirect (via flu infection), direct, and interaction effects. Models were adjusted for demographic, clinical, and social confounders. Sensitivity analyses were conducted.

**Figure:**
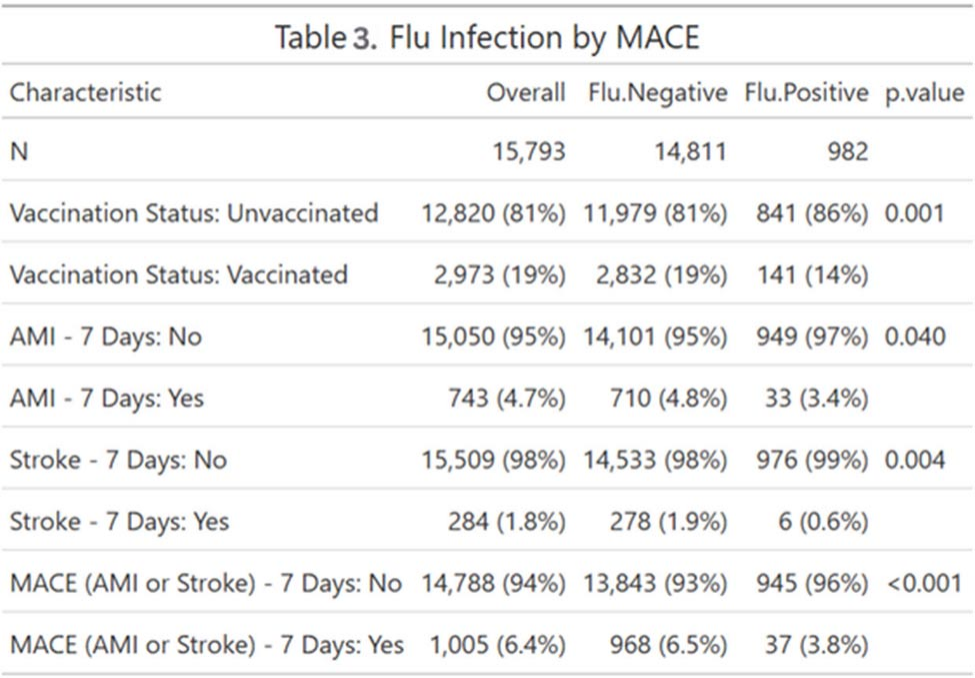
Cross-Tabulation of Vaccination Status, Flu Infection, and 7-Day MACE Outcomes This cross-tabulation shows the 7-day incidence of acute myocardial infarction (AMI), stroke, and composite MACE across four groups defined by vaccination and influenza infection status. The lowest MACE rate (2.1%) occurred among vaccinated, flu-positive individuals, while the highest (6.8%) was among unvaccinated, flu-negative individuals—highlighting potential vaccine-associated cardiovascular protection regardless of flu infection status

**Figure:**
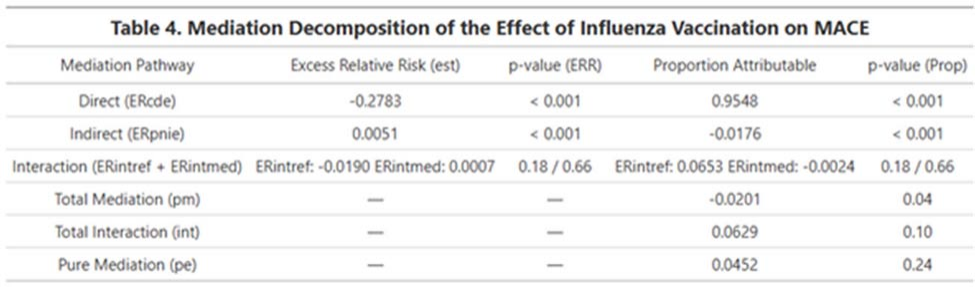
Mediation Decomposition of the Effect of Influenza Vaccination on MACE This table summarizes the results of a causal mediation analysis estimating the excess relative risk (OR − 1) attributable to direct, indirect, and interaction pathways linking vaccine to MACE. Both absolute effects and proportions attributable to each pathway are presented alongside their statistical significance. Notably, the direct effect accounted for the largest proportion of excess risk, while the pure indirect and mediated interaction effects were minimal.

**Results:**

Among 15793 eligible participants, 2,973 (19%) were vaccinated and 982 (6.2%) tested positive for influenza. Overall, 6.4% experienced a MACE event. Vaccinated individuals had lower MACE incidence than the unvaccinated (5.2% vs. 6.6%, *p* = 0.003). Multivariable analysis confirmed a protective effect of vaccination (OR = 0.71, *p* < 0.001). Mediation analysis showed that most of this effect was direct (OR = 0.71, p< 0.001), while the indirect effect via flu infection was negligible (OR = 1.005). A small but statistically significant mediated interaction was observed (OR = 1.008, *p* = 0.04), suggesting minimal modification of flu-related cardiovascular risk by vaccination.

**Conclusion:**

Influenza vaccination was associated with reduced risk of MACE in older adults, primarily through direct non-infectious mechanisms rather than flu prevention.

**Disclosures:**

All Authors: No reported disclosures

